# Motility in *Periweissella* Species: Genomic and Phenotypic Characterization and Update on Motility in *Lactobacillaceae*

**DOI:** 10.3390/microorganisms11122923

**Published:** 2023-12-05

**Authors:** Francesca Fanelli, Marco Montemurro, Daniele Chieffi, Gyu-Sung Cho, Hui-Zhi Low, Frank Hille, Charles M. A. P. Franz, Vincenzina Fusco

**Affiliations:** 1 National Research Council of Italy, Institute of Sciences of Food Production (CNR-ISPA), 70126 Bari, Italy; francesca.fanelli@ispa.cnr.it (F.F.); marco.montemurro@ispa.cnr.it (M.M.); daniele.chieffi@ispa.cnr.it (D.C.); 2 Department of Microbiology and Biotechnology, Max Rubner-Institut, 24103 Kiel, Germany; gyusung.cho@mri.bund.de (G.-S.C.); hui-zhi.low@mri.bund.de (H.-Z.L.); frank.hille@mri.bund.de (F.H.)

**Keywords:** motility, flagella, *fliH*, *fliN*, *Periweissella*, transmission electron microscopy

## Abstract

The genus *Weissella* and the recently described genus *Periweissella*, to which some previously named *Weissella* species have been reclassified as a result of a taxogenomic assessment, includes lactic acid bacteria species with high biotechnological and probiotic potential. Only one species, namely, *Periweissella* (*P*.) *beninensis*, whose type strain has been shown to possess probiotic features, has so far been described to be motile. However, the availability of numerous genome sequences of *Weissella* and *Periweissella* species prompted the possibility to screen for the presence of the genetic determinants encoding motility in *Weissella* and *Periweissellas* spp. other than *P. beninensis*. Herein, we performed a comprehensive genomic analysis to identify motility-related proteins in all *Weissella* and *Periweissella* species described so far, and extended the analysis to the recently sequenced *Lactobacillaceae* spp. Furthermore, we performed motility assays and transmission electron microscopy (TEM) on *Periweissella* type strains to confirm the genomic prediction. The homology-based analysis revealed genes coding for motility proteins only in the type strains of *P. beninensis*, *P. fabalis*, *P. fabaria* and *P. ghanensis* genomes. However, only the *P. beninensis* type strain was positive in the motility assay and displayed run-and-tumble behavior. Many peritrichous and long flagella on bacterial cells were visualized via TEM, as well. As for the *Lactobacillaceae*, in addition to the species previously described to harbor motility proteins, the genetic determinants of motility were also found in the genomes of the type strains of *Lactobacillus rogosae* and *Ligilactobacillus salitolerans*. This study, which is one of the first to analyze the genomes of *Weissella*, *Periweissella* and the recently sequenced *Lactobacillaceae* spp. for the presence of genes coding for motility proteins and which assesses the associated motility phenotypes, provides novel results that expand knowledge on these genera and are useful in the further characterization of lactic acid bacteria.

## 1. Introduction

The genus *Periweissella* was described by Bello et al. in 2022 [1]. They used 16S rRNA gene phylogenetics as well as a phylogenomic analyses (the latter based on the concatenated sequences of 498 core proteins from the genera *Convivina*, *Fructobacillus*, *Leuconostoc*, *Oenococcus* and *Weissella*) to transfer five species that clustered in a separate clade from the genus *Weissella* (*W*.) into the proposed novel genus *Periweissella* (*P.*). These five species included *P. cryptocerci* (basonym *W. cryptocerci* [2]), *P. beninensis* (basonym *W. beninensis* [3]), *P. fabalis* (basonym *W. fabalis* [4]), *P. fabaria* (basonym *W. fabaria* [5]) and *P. ghanensis* (basonym *W. ghanensis* [6]). As is the case for the genus *Weissella*, the novel *Periweissella* genus is also included in the family *Lactobacillaceae*, which was recently revised by Zheng et al. [7], who merged this family together with the *Leuconostocaceae* family. 

Species of the genera *Weissella* and *Periweissella* are widespread in nature and have been isolated from several food matrices, including meat, fish, vegetables and fermented foods, as well as from soil, sewage and the gastrointestinal tracts of humans and animals [8,9]. Despite their probiotic potential, which has recently been demonstrated by several studies [10,11], to date, none of the *Weissella* and *Periweissella* strains are recognized as GRAS (Generally Recognized as Safe) by the Food and Drug Administration (FDA) in the United States of America, nor have they been included in the Qualified Presumption of Safety (QPS) list by the European Food Safety Authority (EFSA). 

Within *Weissella* and *Periweissella* spp., only *P. beninensis* was previously described as being motile [3]. Recently, during the publication process of this manuscript, Qiao et al. [12] reported on the motility of *Periweissella* strains. Bacterial motility, combined with chemotaxis, provides the ability for bacterial dissemination and surface exploration, enabling movement towards favorable conditions to reach resources and allow for surface colonization. These features are also relevant for the probiotic properties of bacterial strains, as motility is important for the initial steps of adhesion to intestinal epithelial cells and biofilm formation [13]. It, furthermore, provides certain advantages regarding survivability within this specific ecological niche and persistence of the microorganisms in the gut mucosa, and is crucial for enabling them to exert their protective effects and for interaction with the host [14].

The recent availability of genome sequences of all *Periweissella* and *Weissella* strains allows for investigations into the presence of genes coding for motility proteins in other species in addition to *P. beninensis*, and if found, these could point towards a potential motile phenotype. In addition, the probiotic potential of some *Weissella* and *Periweissella* strains, recently confirmed by Fanelli et al. [11], also encourages further investigation of functional traits of these strains. This study aimed to perform a comprehensive genomic analysis to identify motility proteins in all *Weissella* and *Periweissella* species described so far, and extended the analysis to the recently sequenced *Lactobacilliaceae* spp. Furthermore, motility assays and transmission electron microscopy (TEM) were carried out on *Periweissella* type strains to confirm our genomic predictions.

## 2. Materials and Methods

### 2.1. Strain Info and Culture Conditions

*P. beninensis* LMG 25373^T^, *P. fabalis* LMG 26217^T^ and *P. fabaria* LMG 24289^T^ were purchased from the Belgian Coordinated Collection of Microorganisms (BCCM/LMG, Ghent, Belgium), while *P. ghanensis* DSM 19935^T^ and *W. diestrammenae* DSM 27940^T^ were purchased from the German Collection of Microorganisms and Cell Cultures (© DSMZ, Braunschweig, Germany). The purity of each strain was confirmed as described in Fanelli et al. [11] via streaking on de Man, Rogosa and Sharpe (MRS; Oxoid, Milan, Italy) agar plates and through microscopic observation. Strains were maintained at −80 °C as pure stock cultures in MRS broth (Oxoid) supplemented with 30% glycerol (*v*/*v*). Strains were routinely grown in MRS broth at 30° under aerobic and static conditions, unless specified otherwise.

### 2.2. Motility Test

Motility was evaluated according to the method described by Shield and Cathcart [15]. *Periweissella* strains were cultured on MRS agar medium (Oxoid). Subsequently, different media, including MRS, PCA (Oxoid) and motility-indole-lysine (MIL) medium [15], were tested to find the one that could best show the strain motility. By using a sterile needle, a colony of each strain was picked and stabbed into the relevant sterile agar medium in tubes to within 1 cm of the bottom. Tubes were incubated at 30 °C until growth became evident. The motility test was considered positive if a diffuse cloud of growth was visibly extending away from the line of inoculation. The test was considered negative if the growth was limited along the inoculation line.

### 2.3. Transmission Electron Microscopy

For transmission electron microscopy, all samples were essentially prepared as described by Sørensen et al. [16], with the modification that 2% (*v*/*v*) glutaraldehyde was used for fixation and 1% (*w*/*v*) uranyl acetate for negative staining. Electron micrographs were taken using a Talos L120C transmission electron microscope (Thermo Fisher Scientific, Eindhoven, The Netherlands) at an accelerating voltage of 80 kV. Digital micrographs were taken using a 4 k × 4 k Ceta camera (Thermo Fisher Scientific).

### 2.4. Comparative Analysis of Flagellar Locus and Motility Proteins 

#### 2.4.1. Identification of Motility Protein Genes in Some *Periweissella* Type Strains

The whole-genome sequencing and the evaluation of the quality of the assemblies were described in Fanelli et al. [17]. The whole-genome shotgun project and GenBank accession number used for the identification of the motility proteins are listed in Supplementary Table S1. Proteins were predicted as described in Fanelli et al. [17] by using the Prokaryotic Genome Annotation Pipeline [18] and PROKKA pipeline [19] implemented in the Galaxy platform (Galaxy Version 1.14.6 + galaxy0; [20]. Functional classification was performed using the RAST server [21]. Genes related to motility and flagellar systems were retrieved through a keyword search within the UniProtID entry list obtained via functional annotation (by using as a query the terms ‘flagella’ and ‘motility’, and by using the RAST server [21]), and then, manually curated.

#### 2.4.2. In Silico Identification of Motile Species in the *Lactobacillaceae* Family

The identification of motile species in the family *Lactobacillaceae* was performed according to the strategy used by Cousin et al. [22], with some modification. Sequences of the predicted filament protein flagellin FliC, the flagellar hook–basal body complex protein FliE, the flagellar motor switch protein FliG, the flagellar biosynthesis protein FlhA and the chemotaxis/stator protein MotA were identified in the proteomes of *Periweissella* spp. Consensus sequences of the selected proteins, generated using EMBOSS Cons [23], were used as query for a TBLASTN search performed against all publicly available *Weissella* species genomes, and then, extended to all recently sequenced and publicly available *Lactobacillaceae* species genomes described after the analysis performed by Cousin et al. [22], according to the taxonomic description amended by Zheng et al. [7], with a cut off E-value of 1 × 10^−10^ and % identity > 30%. Where positive hits were retrieved, motility protein sequences were extracted from the respective proteomes. The homology-based relationship among proteins was determined and confirmed using the BLASTP algorithm on the NCBI site (http://blast.ncbi.nlm.nih.gov/Blast.cgi accessed on 1 October 2023).

#### 2.4.3. Phylogenetic Analysis

The DNA sequences of the 16S rRNA gene of the type strain of each *Lactobacillaceae* species positive for the presence of motility proteins were downloaded from the NCBI GenBank database. Phylogenetic analysis was performed by using (i) the 16S rRNA genes, (ii) a concatenated dataset of all the motility proteins identified in each species assumed as being motile by the in silico analysis, and (iii) concatenated datasets of clusters of motility proteins grouped according to their function or position in the flagellum: chemotaxis proteins (concatenated dataset of CheA, CheB, CheC, CheD, CheW and CheY); basal body–hook proteins (concatenated dataset of FlhA, FlhB, FliE, FliF, FliG, FliH, FliI, FliJ, FliM, FliO, FliP, FliQ, FliR and FliK); filament junction proteins (concatenated dataset of FlgL, FlgK, FliC, FliD and FliS); rod, ring and hook/filament junction proteins (concatenated dataset of FlgB, FlgC, FlgD, FlgE, FlgF and FlgG). Phylogeny was inferred by using an “A la Carte” Mode workflow implemented in the NGphylogeny.fr platform [24] (https://ngphylogeny.fr/workflows/alacarte accessed on 1 October 2023) integrated with MAFFT (version 7.407), BMGE (version 1.12) and FastMe (version 2.1.6.1) with default parameters. The phylogenetic reconstruction was performed using the BIONJ method; the phylogenetic robustness was inferred via a bootstrapping procedure with 1000 replications to obtain the confidence value for the aligned sequence dataset. The trees were graphically generated using iTOL version 6.6 [25]. *Bacillus subtilis* subsp. *subtilis* strain 168 was used as an outgroup.

### 2.5. Brightfield Microscopy

Bacteria grown for 24 h in MRS broth under anaerobic conditions were diluted 1:10 in sterile MRS broth. A total of 200 µL was pipetted into 8-well ibiTreat chambered coverslips (Ibidi, Gräfelfing, Germany) and used for microscopy at 1000× magnification with oil immersion using a Nikon Eclipse TS100 (Nikon, Tokyo, Japan) inverted microscope. The bacteria were allowed to settle for at least 30 min, and videos were recorded using the NIS-Elements D software (version 4.13.03, Nikon). The videos were processed using ImageJ to remove artefacts, and the final videos were compressed to 2× speed and combined.

## 3. Results

### 3.1. Motility Behavior

**Figure 1:**
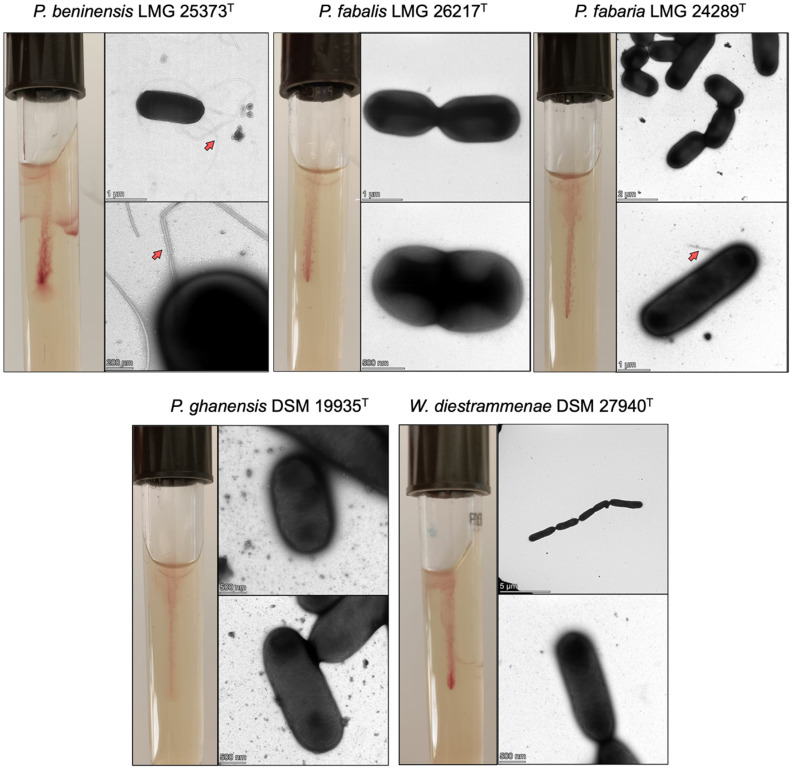
Motility test tubes and TEM of *Periweissella* spp. *W. diestrammenae* DSM 27940^T^ was used as negative control. Flagella are indicated by red arrows.

As reported in the Material and Methods section, different media were tested for the motility assay. *P. beninensis* grew in MRS and PCA, but without the possibility to take a photo and underline its motility, whereas it very clearly showed its motility in MIL. Therefore, MIL was used as a medium for the assay of the motility of all the target strains. Among the four *Periweissella* type strains tested, only the *P. beninensis* type strain was able to grow and spread diffusely within the MIL medium (Figure 1). Negative results were obtained for the *P. fabalis* LMG 26217^T^, *P. fabaria* LMG 24289^T^ and *P. ghanensis* DSM 19935^T^ type strains (Figure 1). *P. beninensis* LMG 25373^T^ was also the only type strain demonstrating overt motility when observed using brightfield microscopy, showing run-and-tumble movements (Supplementary Movie S1).

The other four strains, *P. fabalis* LMG 26217^T^, *P. fabaria* LMG 24289^T^, *P. ghanensis* DSM 19935^T^ and *W. diestrammenae* DSM 27940^T^, did not demonstrate motility beyond vibrations, which did not appear directional in the observed time span (Supplementary Movie S1).

### 3.2. Transmission Electron Microscopy

Figure 1 also shows the electron micrographs of *Periweissella* type strains analyzed in this study. As indicated by the red arrows, only in the *P. beninensis* type strain was it possible to visualize numerous peritrichous and long flagella. In *P. fabaria* LMG 24289^T^, only a rudimental piece of flagellum was visible (red arrow), and such short pieces were visible only in a very minor portion of cells. *W. diestrammenae* DSM 27940^T^ was used as a negative control since its genome was found not to harbor any motility protein genes. 

### 3.3. Comparative Analysis of Flagellar Locus and Motility Proteins 

#### 3.3.1. Presence of Motility Proteins in *Lactobacillaceae*

The homology-based analysis of the *P. beninensis* LMG 25373^T^, *P. fabalis* LMG 26217^T^, *P. fabaria* LMG 24289^T^ and *P. ghanensis* DSM 19935^T^ genomes revealed the presence of motility protein-encoding genes in all four strains. The chemotaxis- and flagella-related proteins identified in *Periweissella* species are listed in Table 1.

**Figure 2:**
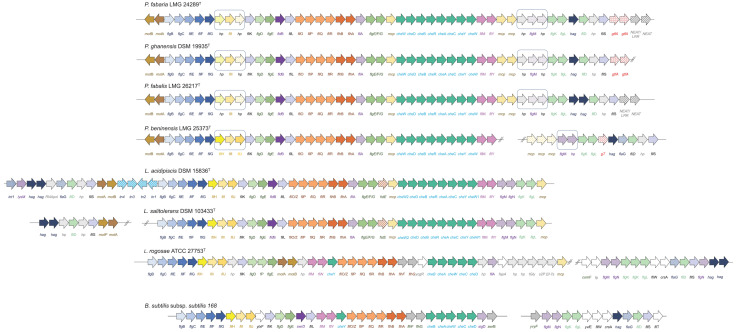
Organization of flagellar locus in *Lactobacillaceae* species. Schematic representation of the flagellar locus in *P. fabalis* LMG 26217^T^, *P. fabaria* LMG 24289^T^, *P. ghanensis* DSM 19935^T^, *P. beninensis* LMG 25373^T^, *L. acidipiscis* DSM 15836^T^, *L. salitolerans* DSM 103433^T^, *L. rogosae* ATCC 27753^T^ and *B. subtilis* subsp. *subtilis* str. 168. Genes are not drawn to scale. Rectangles highlight the *fli*J, *fli*H and *flg*M locus, where the main differences among *P. beninensis* LMG 25373^T^ and the other *Periweissella* species were identified (see text for details).

FlgN was predicted to be encoded in the genomes of all *Periweissella* putative motile species as a hypothetical protein (indicated by *hp* gene near to *flg*M gene in Figure 2). This protein is the flagellum synthesis protein required for the efficient initiation of filament assembly. The FlgN putative homolog proteins identified in *P. beninensis* LMG 25373^T^ (WP_205144167.1) and *P. ghanensis* DSM 19935^T^ (WP_230098022) were classified by InterProScan as having a FlgN-like domain (IPR036679), while no Geno Ontology (GO) term was associated with the putative FlgN homolog of *P. fabalis* LMG 26217^T^ (WP_168721789.1) and *P. fabaria* LMG 24289^T^ (WP_230097033.1), indicating no functional prediction, although the MotifFinder analysis retrieved, for all four hypothetical proteins, the PAFM domain PF05130 associated with the FlgN protein.

Some main differences between the *Periweissella* species positive for the presence of motility proteins could be determined. For instance, the *flaG* gene coding for the FlaG protein (flagella-associated protein) was identified only in *P. beninensis* LMG 25373^T^ (WP_205144163.1); the *fla*G gene was located in the same operon as the *fli*D and *fli*S genes reported in other species (e.g., *Camphylobacter jejuni*), or in the vicinity of the *fli*D operon on the chromosome of many different species of bacteria [26]. 

With respect to the *Weissella* genus, only in the draft genome sequences of *Weissella confusa* L3 and L9 strains, isolated from human feces, were partial sequences of flagellin proteins detected, and they have been deposited under the accession nos. MBS7551942.1, MBS7552003.1, MCQ8173872.1 and MCQ8173814.1. No homolog of the motility proteins listed in Table 1 was identified in the other *Weissella* species, nor in the *P. cryptocerci* type strain, the only remaining species within the *Periweissella* genus so far described. In *Leuconostoc* spp., *Oenococcus* spp., *Convivina* spp. and *Fructobacillus* spp., no motility proteins could be determined. 

In addition to the *Lactobacillaceae* species described by Cousin et al. [22] to harbor motility proteins, they were also identified in the genomes of the recently sequenced *Lactobacillus* (*L*.) *rogosae* ATCC 27753^T^ (and identical to those retrieved from the genome of the *L. rogosae* isolate avicel METABAT 177 assembled from the human gut microbiome) and *Ligilactobacillus salitolerans* DSM 103433^T^ (Figure 2).

#### 3.3.2. Comparative Analysis of Motility Locus in *Periweissella* Species

The organization of the flagellar locus of the *Periweissella* type strains analyzed in this study is illustrated in Figure 2, while motility protein IDs are listed in Table 1. 

In *P. fabaria* LMG 24289^T^, *P. fabalis* LMG 26217^T^ and *P. ghanensis* DSM 19935^T^, the motility gene locus was annotated on a unique contig, while in *P. beninensis* LMG 25373^T^ the *fliD* operon (*fliD* operon; following nomenclature of corresponding operons in *Bacillus subtilis* and *Salmonella enterica* Typhimurium, containing the genes *fliD*, *fliS* and *fliT*) [27,28] was annotated in a separated contig (Figure 2). The organization of the flagellar operon was conserved in the *Periweissella* species, while the gene content and arrangement within the *fliD* operon showed some differences: (i) Downstream of the FliS coding gene in *P. fabaria* LMG 24289^T^ and *P. ghanensis* DSM 19935^T^ were two genes (*gtfA*) annotated to code for a UDP-N-acetylglucosamine-peptide N-acetylglucosaminyltransferase GtfA subunit; (ii) in *P. fabaria* LMG 24289^T^ and *P. fabalis* LMG 26217^T^, there were two genes coding for two hypothetical proteins, both harboring a NEAT domain, which was found in a group of iron-regulated surface determinant proteins. In addition, one of these was predicted to have an internaline surface protein domain with a leucine-rich repeat (LRR) that allowed it to provide a structural framework for the formation of protein–protein interactions. (iii) The *fliD* operon of *P. fabalis* LMG 26217^T^ also harbored two *hag* genes coding for the two flagellins FliC1 and FliC2, as previously described, while only one *hag* gene was present in the other *Periweissella* spp. (iv) In the *fliD* operon in *P. beninensis* LMG 25373^T^ one gene was annotated that codes for FlaG and one for a glycosyltransferase located between the *flg*L and *hag* gene, respectively, and three methyl-accepting chemotaxis protein (Mcp)-encoding genes were located upstream of the *flg*M operon (Figure 2), which exceeded the count observed in the loci of the other *Periweissella* species by one. 

Another difference in the motility genes of the different *Periweissella* species was that in *P. beninensis* LMG 25373^T^, there were the putative homologs of proteins, annotated as FliH and FliJ (WP_205144100.1 and WP_205144102.1, respectively; Table 1 and boxed parts in Figure 2). These were two cytoplasmic ATPase complex proteins that are part of the flagellum export apparatus [29]. Meanwhile, in *P. fabalis* LMG 26217^T^, *P. fabaria* LMG 24289^T^ and *P. ghanensis* DSM 19935^T^, they were annotated as hypothetical proteins. The similarity among these hypothetical proteins and the respective homolog in *P. beninensis* LMG 25373^T^ was, on average, ca. 35%, while between *P. fabaria* LMG 24289^T^ and the respective homolog in *P. ghanensis* DSM 19935^T^, it reached 99%, and between *P. fabalis* LMG 26217^T^ and the respective homolog in *P. fabaria* LMG 24289^T^ and *P. ghanensis* DSM 19935^T^, it was around 75–80%. The MotifFinder pipeline predicted, in the three hypothetical proteins homologous to FliH of *P. fabalis* LMG 26217^T^, *P. fabaria* LMG 24289^T^ and *P. ghanensis* DSM 19935^T^, the PFAM domain PF02108 associated with the flagellar assembly protein FliH, and in all three hypothetical protein homologs of FliJ, the PFAM domain PF02050 associated with the flagellar FliJ protein.

As mentioned before, a difference in the motility genes among the *Periweissella* species is that only in *P. fabalis* LMG 26217^T^ was a second *hag (fliC2)* gene coding for the flagellin FliC2 annotated in the flagellar operon (WP_168721785.1): the predicted FliC2 shares 75% similarity with FliC1 (WP_168721786.1), and 78% and 65% with the homologs in *P. fabaria* LMG 24289^T^ (WP_230097036.1) and *Ligilactobacillus ruminis* AM12-58A (WP_270413121.1), respectively. In *Ligilactobacillus ruminis* AM12-58A, the *fli*C2 flagellin gene was 98% identical at nucleotide level to the *fli*C1 gene encoded in the same strain; however, gene expression studies showed that only *fli*C2 was expressed at a high level in the motile strains [30]. Multiple flagellin proteins were also described to occur in several species, such as *Sinorhizobium meliloti*, *Rhizobium leguminosarum*, *Agrobacterium tumefaciens* and *Caulobacter crescentus*, the latter of which has a single polar flagellum and six flagellin proteins [31,32,33,34].

The main differences between the flagellar loci of the non-motile *Periweissella* strains *P. fabalis* LMG 26217^T^, *P. fabaria* LMG 24289^T^ and *P. ghanensis* DSM 19935^T^ with respect to the motile *P. beninensis* LMG 25373^T^ thus appears to be the absence of the *flaG* gene and the presence of hypothetical genes showing low homology to the corresponding (*fliH*, *fliJ*, *flgN*) genes. The identity of the hypothetical genes was validated using HHpred, which allows for the detection of remote homologs by comparing protein profiles based on Hidden Markov Models rather than only relying on sequence homology. The hypothetical protein sequences were screened against the RCSB Protein Data Bank database (https://www.rcsb.org/ accessed on 1 October 2023) and yielded hits for FliH, FliJ and FlgN, respectively. 

The ATPase complex promoting the flagellar protein export machinery is composed of FliH, FliJ and FliI, where the latter exhibits enzymatic ATPase activity. The characteristic Walker A (GxxxxGK(S/T)—where x is any amino acid) and Walker B (hhhhDE—where h is any hydrophobic amino acid) motifs found in ATPases were detected in all *Periweissella* homologs (Supplementary Figure S1), suggesting that all homologs are catalytically active. Surprisingly, there are major differences in the conservation of the three proteins forming the ATPase complex between the analyzed *Periweissella* species. While FliI is highly conserved between the here determined non-motile species *P. fabaria*, *P. fabalis* and *P. ghanensis* (protein similarity 98.2–98.5%) and rather well conserved in *P. beninensis* (protein similarity 72.4–72.9%), the other two proteins forming the ATPase complex (FliH and FliJ) display a decreased homology between *P. beninensis* and the three herein determined as non-motile *Periweissella* species. Among only the latter species, FliH displays an amino acid sequence similarity of 71.5% and FliJ of 79.2–81.9%. Compared to *P. beninensis*, protein similarity decreases to 25.9–33.2% for FliH and 31.7–33.7% for FliJ, thus demonstrating unequal conservation of the ATPase complex. Interestingly, these genes are all present and share higher homology in all the other motile lactic acid bacteria examined in this study (*L. acidipisces* DSM 15836^T^, *L. salitotolerans* DSM 103433^T^ and *L. rogosae* ATCC 27753^T^ (Figure 2)), the only exception being that the *flaG* gene is not present in the motility locus of *L. salitotolerans* DSM 103433^T^. On the other hand, the *fliD* gene encoding the filament cap protein, which is a protein for chaperoning and sorting flagellin (FliC) proteins after they traverse the hollow filament and exit the growing flagellum tip [35], was only found in the flagellar loci of the *P. fabalis* LMG 26217^T^, *P. fabaria* LMG 24289^T^ and *P. ghanensis* DSM 19935^T^ strains, but not in the corresponding locus of the *P. beninensis* strain LMG 25373^T^ (Figure 2). As shown in Figure 2, the content and organization of the flagellar locus in *Periweissella* species compared to other *Lactobacillaceae*, such as the *L. acidipiscis* type strain and *L. salitolerans* type strain, is quite different. In *L. salitolerans* DSM 103433^T^, *motA* and *motB* genes were located within the *fli*D operon, which comprised two flagellin genes and was annotated in a separate contig with respect to the flagellar operon. Also, in *Lactobacillus rogosae* ATCC 27753^T^, the *fli*D operon was annotated on a separate contig with respect to the flagellar operon, which misses *flb*D, *fli*K and *fli*L and harbors the *fap*A gene coding for the flagellar assembly protein FapA, and ycgR coding for PliZ, a cyclic-di-GMP (c-di-GMP) associated with flagellar and pili-based motility. In the *L. acidipiscis* type strain *fli*D operon, there are four genes annotated as transposase, *mot*B and *motA* genes, the *fliS* and *fliD* gene interspaced by a gene coding for a hypothetical protein, the *fla*G gene, one RNA polymerase-coding gene, two flagellin *hag* genes, a gene coding for the peptidoglycan-binding protein LysM and one integrase.

#### 3.3.3. Phylogeny of the Motility Proteins in the *Lactobacillaceae* Species

**Figure 3:**
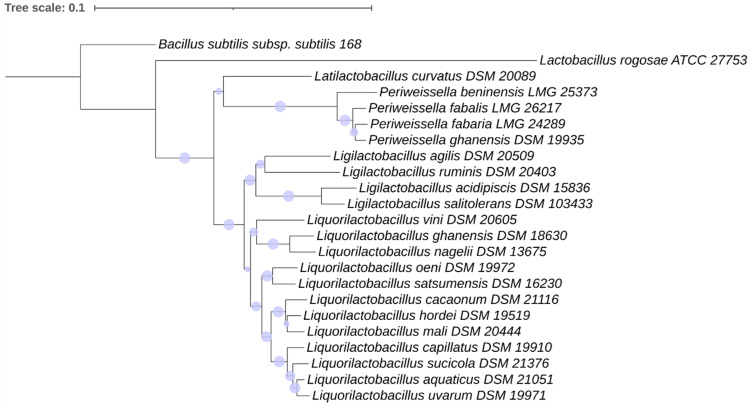
16S rRNA gene-based phylogeny of *Lactobacillaceae* species. Phylogeny was performed using the neighbor-joining method; phylogenetic robustness was inferred via a bootstrapping procedure from 1000 replications to obtain the confidence value for the aligned sequence dataset. *Bacillus subtilis* subsp. *subtilis* str. 168 was used as an outgroup. The tree was drawn to scale, with branch lengths measured as the number of substitutions per site. Scaled circles are representative of bootstrap values.

**Figure 4:**
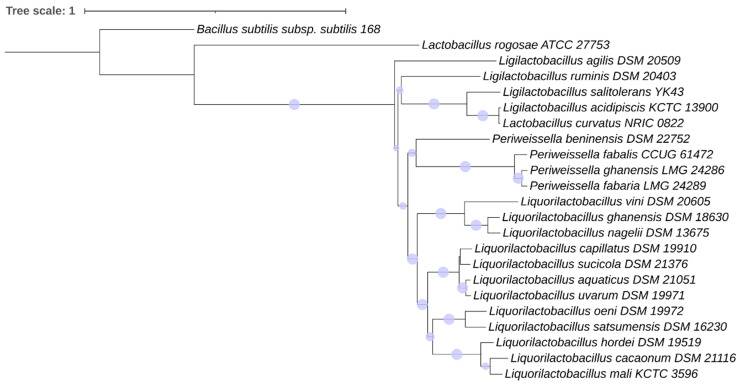
Phylogenetic analysis of motility proteins in *Lactobacillaceae* species. The tree is based on the concatenated dataset of motility proteins (see text for details). Phylogenetic robustness was inferred via a bootstrapping procedure from 1000 replications to obtain the confidence value for the aligned sequence dataset. *Bacillus subtilis* subsp. *subtilis* str. 168 was used as an outgroup. The tree was drawn to scale, with branch lengths measured as the number of substitutions per site. Scaled circles are representative of bootstrap values.

Figure 3 and Figure 4 show the concordant phylogeny of 16S rRNA genes and motility proteins in the *Lactobacillaceae* family. *P. beninensis* LMG 25373^T^, *P. fabalis* LMG 26217^T^, *P. fabaria* LMG 24289^T^ and *P. ghanensis* DSM 19935^T^ were always clustered in one separate clade, with *P. fabaria* LMG 24289^T^ and *P. ghanensis* DSM 19935^T^ being on the same node, and slightly divergent from *P. fabalis* LMG 26217^T^ and more divergent from *P. beninensis* LMG 25373^T^ (Figure 3 and Figure 4). *P. beninensis* LMG 25373^T^ was clustered together with the other *Periweissella* species in the phylogenetic tree of the concatenated dataset of all the motility proteins, and of the groups of chemotaxis proteins, filament junction proteins, and rod–ring–hook proteins, although, interestingly, it was also grouped apart from these, showing a clear difference in the motility gene content outlined above. Furthermore, in a few cases, the phylogeny of individual proteins, such as CheB, CheR, CheC, CheD, FlgB, FlgC, etc., as well as of those occurring in the cluster of the basal body and hook proteins (shown in Supplementary Figure S2B), underlines a higher proximity of *P. beninensis* LMG 25373^T^ with species outside of the *Periweissella* genus.

The phylogenetic analysis of the motility proteins mostly overlapped with the 16S rRNA gene sequence phylogeny, and in both trees (Figure 3 and Figure 4), almost all the *Lactobacillaceae* species clustered in the same clade: (1) *L. capillatus*, *L. sucicola*, *L. acquaticus* and *L. uvarum*; (2) *L. cacaonum*, *L. hordei* and *L. mali*; (3) *L. oeni* and *L. satsumensis*; (4) *L. ghanensis*, *L. nagelii* and *L. vini*; (5) *L. acidipiscis* and *L. salitolerans*; (6) *P. beninensis*, *P. ghanensis*, *P. fabalis* and *P. fabaria*.

*L. rogosae* ATCC 27753^T^ (as well as *L. rogosae* isolate avicel METABAT 177) was phylogenetically apart from the other *Lactilobacillaceae* species in each motility protein analyzed, as well as in relation to the 16S rRNA gene sequence phylogeny (Figure 3 and Figure 4).

## 4. Discussion

The bacterial flagellum is organized into three basic components [36,37]: (1) The basal body, anchored in the cell membrane and comprising a rotary motor, connected to a rod, which powers the flagellum, the export apparatus machinery, and bearing structures. (2) The hook [38], which functions like a universal joint and is a tubular helical structure made via the polymerization of multiple copies of a protein, FlgE; its length is controlled by FliK. The hook transmits the torque produced by the motor to the (3) filament, which is the propeller of the flagellum, with FliC as the core unit and FliD acting as a cap. The junction between the hook and the filament is the charger of FlgL and FlgK.

The flagellar apparatus in *Salmonella enterica* subsp. *enterica* serovar Typhimurium is one of the best characterized thanks to the work by Minamino and coworkers [29], and the crystal structures of most of the protein complexes that comprise the flagellar apparatus have been resolved and deposited in the RCSB Protein Data Bank. Both FliH and FliJ, which are also present in the genetic locus of motile *P. beninensis* LMG 25373^T^, have been described to be part of the FliH(12)-FliI(6)-FliJ(1) cytoplasmic ATPase export ring complex, which acts as an active protein transporter. Its structure resembles the cytoplasmic part of F_O_F_1_ ATP synthase, a rotary motor that couples proton (H^+^) flow through F_O_ with ATP synthesis by F_1_ [39]. We found that the conservation of the subunits of the ATPase involved in protein export is dissimilar between the *Periweissella* strains. While FliI is rather highly conserved, FliH and FliJ display considerably lower sequence homology between *P. beninensis* and the three non-motile species. This could indicate that the complex might not form properly in the non-motile species due to the high evolutionary divergence, resulting in an impaired flagellar export mechanism, and thus, could explain the difference in motility.

In addition to that, FlgN was also poorly conserved. FlgN is one of the flagellar chaperones, together with FliS and FliT, specific to FlgK and FlgL, which, in *Bacillus subtilis*, is required for flagellum-based motility [40]. The crystal structure of FlgN in *Salmonella* was solved in 2016 by Kinoshita et al. [41] and consists of three helices. The authors showed that a conformational change in FlgN with rearrangements of α-helices is critical for the association and dissociation of FlgK [41]. Minamino et al. [42] demonstrated that FlgN promotes the docking of FlgK and FlgL to the FlhA platform of the export gate complex to facilitate the rapid and efficient export of these proteins [43]. The impairing of this activity reduces the secretion levels of FlgK and FlgL, resulting in a considerable reduction in the probability of filament formation at the tip of the hook–basal body [44]. In addition to its chaperone activity, FlgN interacts with FlhA, which is the export engine of the apparatus, generating a conformational change in FlhA that allows it to function as a Na^+^ channel.

The *flaG* gene was identified only in *P. beninensis* LMG 25373^T^. FlaG is one of the flagellar proteins that are actually absent in well-studied model organisms such as *Escherichia coli* and *Salmonella enterica* [45,46], but has been detected and characterized in other motile species such as *Aeromonas caviae*, *Campylobacter jejuni*, *Pseudomonas aeruginosa* and *Pseudomonas fluorescens* [47,48,49]. Divergence of the protein sequences and mutational behaviors in different species suggested that FlaG had a possible role in regulating the export of flagellin proteins, filament length and the number or the expression of other flagellin genes [50,51,52].

Our study shows that hypothetical proteins with putative homology to FliH, FliJ and FlgN in the three non-motile *Periweissella* species have only low similarity (35–41%) when compared to those comprising the flagellar locus of *P. beninensis* LMG 25373^T^. As FliH and FliJ are essential for the export of flagellar proteins, it is possible that a low homology of counterpart proteins in the non-motile strains implies that the flagellar components cannot be sufficiently exported for flagellar biosynthesis. Similarly, FlgN is a flagellum synthesis protein and is required for the export of the hook/filament junction proteins FlgK and FlgL. If this is absent or nonfunctional, again, this could prevent the assembly of a functional flagellum, although we have evidence that in *P. fabaria* LMG 24289^T^, a partial flagellum could possibly be assembled, as seen in the results of electron microscopy (Figure 1).

Despite the fact that *P. fabalis* LMG 26217^T^, *P. fabaria* LMG 24289^T^ and *P. ghanensis* DSM 19935^T^ possess a complete flagellar operon, motility could be demonstrated only for *P. beninensis* LMG 25373^T^. It is very clear from live cell video microscopy that *P. beninensis* LMG 25373^T^ bacteria are remarkably motile compared to the other *Periweisella* species (Supplementary Movie S1).

Our data are in agreement with previous papers describing *Periweissella* spp. In particular *P. fabalis* [4] and *P. fabaria* [5] were described as not motile, and for *P. ghanensis*, gliding motility was not observed [6], while, conversely, *P. beninensis* [3] was defined as motile with peritrichous flagella that can occur singly, in pairs or in short chains.

A recent paper published by Qiao et al. [12] confirmed the presence of the flagellar operon in *P. beninensis*, *P. fabalis* and *P. fabaria*, and the authors described all these species as motile. However, the only phenotypic assessment to prove their assumption was the stab test in semisolid MRS agar medium, which, for us, was not as adequately informative for assessing motility compared to using MIL medium. Furthermore, surprisingly, *P. beninensis* was not able to grow under the condition tested by Qiao et al. [12]. In our work, the TEM experiments showed that with the exception of the *P. beninensis* type strain, the other *Periweissella* species were not able to assemble any visible flagellar apparatus under the tested condition. Only in *P. fabaria* LMG 24289^T^ was a rudimentary piece of flagellum visible, and very few pieces of flagella could be found only in a very minor portion of cells. This was supported by microscopic observations, which showed that only *P. beninensis* had high motility. Hypothetically the genomic differences reported here in the flagellar operons of the other *Periweissela* when compared to *P. beninensis* LMG 25373^T^ might lead to a lower expression of flagellar proteins under the condition that we tested in this study. This should, however, be investigated for media and test conditions in further studies.

It is interesting to note that, in addition to other *Weissella* species, in the *P. cryptocerci* type strain, no motility proteins could be determined. Previous work by our group [11,17] demonstrated that *P. beninensis* LMG 25373^T^ displayed the best probiotic behavior, with the highest adhesion capacity, as well as a varied carbohydrate utilization profile, and was the only one capable of using d-galactose. Although motility is not essential for gut colonization, it might provide certain advantages in survivability and persistence for these organisms in the gut mucosa, and favor colonization in the gastrointestinal tract, as occurred for motile lactobacilli compared to not-motile ones [53].

Flagella are composed, together with pili, of surface layer proteins (SLPs), capsular polysaccharide (CPS), lipoteichoic acid and lipopolysaccharide, the surface components of probiotics that constitute microbe-associated molecular patterns (MAMPs) [54], which can specifically bind to pattern recognition receptors and regulate nuclear factor kappa B, mitogen-activated protein kinases, peroxisome proliferator-activated receptor gamma, and other signaling pathways in intestinal epithelial cells [55], in addition to activating the cellular protease-dependent signaling cascade to produce a variety of cytokines and chemokines that alleviate inflammation and enhance intestinal epithelial function [56]. In the probiotic *E. coli* Nissle 1917, the flagellum is not just responsible for motility and capable of inducing the production of human antimicrobial peptide β-defensin 2, but is the major adhesin mediating binding to human mucus [57], which enables this probiotic strain to compete efficiently for binding sites on host tissue with several pathogenic bacteria. The ability of *P. beninensis* LMG 25373^T^ to assemble a functional flagellum thus confirms the distinctiveness of this strain with respect to the other *Periweissella* type strains analyzed, and suggests a correlation with optimal probiotic activity, in particular related to its adhesion ability, that was recently demonstrated in vitro by Fanelli and coworkers [17].

## 5. Conclusions

The comprehensive genomic analysis herein carried out to detect the genetic determinants of motility in *Weissella*, *Periweissella* and the recently sequenced *Lactobacillaceae* spp. allowed us to detect genes coding for motility proteins only in the type strains of *P. beninensis*, *P. fabalis*, *P. fabaria* and *P. ghanensis* genomes, as well as in the recently genome-sequenced type strains of *Lactobacillus rogosae* and *Ligilactobacillus salitolerans*. Among the *Weissella* and *Periweissella* type strains analyzed in this study, only the *P. beninensis* type was positive in the motility assay, and its many peritrichous and long flagella were visualized via TEM, while, despite the type strains of *P. fabalis*, *P. fabaria* and *P. ghanensis* possessing a complete flagellar operon, no motility was observed in these strains under the conditions used in this study, and only the *P. fabaria* type strain showed a rudimentary piece of flagellum on few cells. The type strain of *P. beninesis* showed the highest in vitro adhesion capacity and resistance to simulated gastrointestinal digestion [17] (Fanelli et al., 2023), indicating a possible correlation between the motile behavior and the probiotic potential of this strain.

This study provides novel results that are useful in the further characterization of these genera.

## Figures and Tables

**Table 1:** Flagellar and chemotaxis proteins predicted in *Periweissella* strains ^a^.

Product	*P. beninensis* LMG 25373^T^	*P. fabalis* LMG 26217^T^	*P. fabaria* LMG 24289^T^	*P. ghanensis* DSM 19935^T^
flagellar motor protein MotB	WP_205144093.1 KAK10_03145	WP_168721826.1 KAR41_09605	WP_230096995.1 KAR50_04000	WP_230096995.1 KAR53_05550
MotA/TolQ/ExbB proton channel family protein	WP_205144094.1 KAK10_03150	WP_168721825.1 KAR41_09610	WP_230096996.1 KAR50_04005	WP_230097992.1 KAR53_05545
flagellar basal body rod protein FlgB	WP_205144095.1 KAK10_03155	WP_168721824.1 KAR41_09615	WP_230096997.1 KAR50_04010	WP_230096997.1 KAR53_05540
flagellar basal body rod protein FlgC	WP_205144096.1 KAK10_03160	WP_168721823.1 KAR41_09620	WP_230096998.1 KAR50_04015	WP_230097993.1 KAR53_05535
flagellar hook–basal body complex protein FliE	WP_205144097.1 KAK10_03165	WP_210726990.1 KAR41_09625	WP_230096999.1 KAR50_04020	WP_230097994.1 KAR53_05530
flagellar M-ring protein FliF	WP_205144098.1 KAK10_03170	WP_168721822.1 KAR41_09630	WP_230097000.1 KAR50_04025	WP_230097995.1 KAR53_05525
flagellar motor switch protein FliG	WP_205144099.1 KAK10_03175	WP_168721821.1 KAR41_09635	WP_230097001.1 KAR50_04030	WP_230097996.1 KAR53_05520
flagellar assembly protein FliH	WP_205144100.1 KAK10_03180	WP_168721820.1 KAR41_09640 ^b^	WP_230097002.1 KAR50_04035 ^b^	WP_230097997.1 KAR53_05515 ^b^
flagellar protein export ATPase FliI	WP_205144101.1 KAK10_03185	WP_168721819.1 KAR41_09645	WP_230097003.1 KAR50_04040	WP_230097998.1 KAR53_05510
flagellar export protein FliJ	WP_205144102.1 KAK10_03190	WP_168721818.1 KAR41_09650 ^b^	WP_230096704.1 KAR50_04045 ^b^	WP_230097999.1 KAR53_05505 ^b^
flagellar hook–length control protein FliK	WP_205144103.1 KAK10_03195	WP_168721817.1 KAR41_09655	WP_230097005.1 KAR50_04050	WP_230098000.1 KAR53_05500
flagellar basal body rod modification protein FlgD	WP_205144104.1 KAK10_03200	WP_210726989.1 KAR41_09660	WP_230097006.1 KAR50_04055	WP_230097006.1 KAR53_05495
flagellar hook–basal body complex protein FlgE	WP_205144105.1 KAK10_03205	WP_168721816.1 KAR41_09665	WP_230097007.1 KAR50_04060	WP_230098001.1 KAR53_05490
flagellar FlbD family protein	WP_205144106.1 KAK10_03210	WP_168721815.1 KAR41_09670	WP_230097008.1 KAR50_04065	WP_230098002.1 KAR53_05485
flagellar basal body-associated FliL family protein	WP_205144107.1 KAK10_03215	WP_168721814.1 KAR41_09675	WP_230097009.1 KAR50_04070	WP_230098003.1 KAR53_05480
flagellar biosynthetic protein FliO	WP_205144108.1 KAK10_03220	WP_168721813.1 KAR41_09680	WP_230097010.1 KAR50_04075	WP_230098004.1 KAR53_05475
flagellar type III secretion system pore protein FliP	WP_205144109.1 KAK10_03225	WP_168721812.1 KAR41_09685	WP_230097011.1 KAR50_04080	WP_230097011.1 KAR53_05470
flagellar biosynthesis protein FliQ	WP_205144110.1 KAK10_03230	WP_168721811.1 KAR41_09690	WP_230097012.1 KAR50_04085	WP_230097012.1 KAR53_05465
flagellar biosynthetic protein FliR	WP_205144111.1 KAK10_03235	WP_168721810.1 KAR41_09695	WP_230097013.1 KAR50_04090	WP_230098005.1 KAR53_05460
flagellar biosynthesis protein FlhB	WP_205144112.1 KAK10_03240	WP_168721809.1 KAR41_09700	WP_230096714.1 KAR50_04095	WP_230098006.1 KAR53_05455
flagellar biosynthesis protein FlhA	WP_205144113.1 KAK10_03245	WP_168721808.1 KAR41_09705	WP_230097015.1 KAR50_04100	WP_230098007.1 KAR53_05450
FliA/WhiG family RNA polymerase sigma factor	WP_205144114.1 KAK10_03250	WP_168721807.1 KAR41_09710	WP_230097016.1 KAR50_04105	WP_230098008.1 KAR53_05445
flagellar hook–basal body complex protein FlgEFG	WP_205144115.1 KAK10_03255	WP_168721806.1 KAR41_09715	WP_230097017.1 KAR50_04110	WP_230098009.1 KAR53_05440
flagellar hook–basal body protein FlgEFG	WP_205144116.1 KAK10_03260	WP_168721805.1 KAR41_09720	WP_230097018.1 KAR50_04115	WP_230098010.1 KAR53_05435
methyl-accepting chemotaxis protein	WP_205144117.1 KAK10_03265	WP_168721804.1 KAR41_09725	WP_230097019.1 KAR50_04120	WP_230098011.1 KAR53_05430
chemotaxis protein CheW	WP_205144118.1 KAK10_03270	WP_168721803.1 KAR41_09730	WP_230097020.1 KAR50_04125	WP_230097020.1 KAR53_05425
chemotaxis protein CheD	WP_205144119.1 KAK10_03275	WP_168721802.1 KAR41_09735	WP_230097021.1 KAR50_04130	WP_230098012.1 KAR53_05420
chemotaxis-specific protein-glutamate methyltransferase CheB	WP_205144120.1 KAK10_03280	WP_168721801.1 KAR41_09740	WP_230097022.1 KAR50_04135	WP_230098013.1 KAR53_05415
protein-glutamate O-methyltransferase CheR	WP_205144121.1 KAK10_03285	WP_168721800.1 KAR41_09745	WP_230097057.1 KAR50_04140	WP_230098035.1 KAR53_05410
chemotaxis protein CheA	WP_205144122.1 KAK10_03290	WP_168721799.1 KAR41_09750	WP_230097023.1 KAR50_04145	WP_230098014.1 KAR53_05405
chemotaxis protein CheC	WP_205144123.1 KAK10_03295	WP_168721798.1 KAR41_09755	WP_230097024.1 KAR50_04150	WP_230098015.1 KAR53_05400
response regulator	WP_205144124.1 KAK10_03300	WP_168721797.1 KAR41_09760	WP_230097025.1 KAR50_04155	WP_230097025.1 KAR53_05395
chemotaxis protein CheW	WP_205144125.1 KAK10_03305	WP_168721796.1 KAR41_09765	WP_230097026.1 KAR50_04160	WP_230097026.1 KAR53_05390
flagellar motor switch protein FliM	WP_205144126.1 KAK10_03310	WP_168721795.1 KAR41_09770	WP_230097027.1 KAR50_04165	WP_230098016.1 KAR53_05385
flagellar motor switch phosphatase FliY	WP_205144127.1 KAK10_03315	WP_168721794.1 KAR41_09775	WP_230097028.1 KAR50_04170	WP_230098017.1 KAR53_05380
methyl-accepting chemotaxis protein	WP_205144169.1 KAK10_03445	WP_168721793.1 KAR41_09780	WP_230097029.1 KAR50_04175	WP_230098018.1 KAR53_05375
methyl-accepting chemotaxis protein	WP_239517065.1 KAK10_03450	WP_168721792.1 KAR41_09785	WP_230097030.1 KAR50_04180	WP_230098019.1 KAR53_05370
methyl-accepting chemotaxis protein	WP_239517064.1 KAK10_03455	na	na	na
flagellar biosynthesis anti-sigma factor FlgM	WP_205144168.1 KAK10_03460	WP_168721790.1 KAR41_09795	WP_230097032.1 KAR50_04190	WP_230098021.1 KAR53_05360
Flagella synthesis protein FlgN	WP_205144167.1 ^b^ KAK10_03465	WP_168721789.1 ^b^ KAR41_09800	WP_230097033.1 ^b^ KAR50_04195	WP_230098022.1 ^b^ KAR53_05355
flagellar hook-associated protein FlgK	WP_205144166.1 KAK10_03470	WP_168721788.1 KAR41_09805	WP_230097034.1 KAR50_04200	WP_230098023.1 KAR53_05350
flagellar hook-associated protein FlgL	WP_205144165.1 KAK10_03475	WP_168721787.1 KAR41_09810	WP_230097035.1 KAR50_04205	WP_230098024.1 KAR53_05345
Flagellin FliC1	WP_205144164.1 KAK10_03485	WP_168721786.1 KAR41_01540	WP_230097036.1 KAR50_04210	WP_230098025.1 KAR53_05340
FliC2	na	WP_168721785.1 KAR41_01545	na	na
flagellar protein FlaG	WP_205144163.1 KAK10_03490	na	na	na
flagellar filament-capping protein FliD	WP_205144162.1 KAK10_03495	WP_168721784.1 KAR41_06490	WP_230097037.1 KAR50_04215	WP_230098026.1 KAR53_05335
flagellar export chaperone FliS	WP_205144160.1 KAK10_03505	WP_168721782.1 KAR41_01555	WP_230097038.1 KAR50_04225	WP_230098028.1 KAR53_05325

^a^ in the columns, protein ID and locus tag are indicated; ^b^ annotated as hypothetical protein; na = not annotated.

## Data Availability

All data generated or analyzed during this study are included in this published article and its Supplementary Material.

## Supplementary Materials

The following supporting information can be downloaded at: https://www.mdpi.com/article/10.3390/microorganisms11122923/s1, Table S1. List of *Lactobacillaceae* type strains and WGS reference accession used in this study. Movie S1. Brightfield microscopy videos (2× speed) depicting run-and-tumble motility in *P. beninensis* LMG 25373^T^ in comparison to the lack thereof in other *Periweissella* and *Weissella* strains. Figure S1. Protein sequence alignments of different *Periweissella* FliI homologs. Conservation of each residue is represented by colored bars, where tall yellow bars indicate a conservation of 100%. Amino acids that constitute the putative Walker A and Walker B motifs are labeled in red. Figure S2. Phylogenetic tree of motility proteins concatenated and grouped according to their function or position in the flagellum. (A) Chemotaxis proteins; (B) basal body–hook proteins; (C) filament junction proteins; (D) rod, ring and hook/filament junction proteins. Phylogenetic robustness was inferred via a bootstrapping procedure from 1000 replications to obtain the confidence value for the aligned sequence dataset. *Bacillus subtilis* subsp. *subtilis* str. 168 was used as an outgroup. The trees were drawn to scale, with branch lengths measured as the number of substitutions per site. Scaled circles are representative of bootstrap values.

## References

[1] Bello S., Rudra B., Gupta R.S. (2022). Phylogenomic and comparative genomic analyses of *Leuconostocaceae* species: Identification of molecular signatures specific for the genera *Leuconostoc*, *Fructobacillus* and *Oenococcus* and proposal for a novel genus *Periweissella* gen. nov. Int. J. Syst. Evol. Microbiol..

[2] Heo J., Hamada M., Cho H., Weon H.Y., Kim J.S., Hong S.B., Kim S.J., Kwon S.W. (2019). *Weissella cryptocerci* sp. nov., isolated from gut of the insect *Cryptocercus kyebangensis*. Int. J. Syst. Evol. Microbiol..

[3] Padonou S.W., Schillinger U., Nielsen D.S., Franz C.M.A.P., Hansen M., Hounhouigan J.D., Nago M.C., Jakobsen M. (2009). *Weissella beninensis* sp. nov., a motile lactic acid bacterium from submerged cassava fermentations, and emended description of the genus *Weissella*. Int. J. Syst. Evol. Microbiol..

[4] Snauwaert I., Papalexandratou Z., De Vuyst L., Vandamme P. (2013). Characterization of strains of *Weissella fabalis* sp. nov. and Fructobacillus tropaeoli from spontaneous cocoa bean fermentations. Int. J. Syst. Evol. Microbiol..

[5] De Bruyne K., Camu N., De Vuyst L., Vandamme P. (2010). *Weissella fabaria* sp. nov., from a Ghanaian cocoa fermentation. Int. J. Syst. Evol. Microbiol..

[6] De Bruyne K., Camu N., Lefebvre K., De Vuyst L., Vandamme P. (2008). *Weissella ghanensis* sp. nov., isolated from a Ghanaian cocoa fermentation. Int. J. Syst. Evol. Microbiol..

[7] Zheng J., Wittouck S., Salvetti E., Franz C.M.A.P., Harris H.M.B., Mattarelli P., O’Toole P.W., Pot B., Vandamme P., Walter J. (2020). A taxonomic note on the genus *Lactobacillus*: Description of 23 novel genera, emended description of the genus *Lactobacillus* Beijerinck 1901, and union of *Lactobacillaceae* and *Leuconostocaceae*. Int. J. Syst. Evol. Microbiol..

[8] Fusco V., Quero G.M., Cho G.S., Kabisch J., Meske D., Neve H., Bockelmann W., Franz C.M.A.P. (2015). The genus *Weissella*: Taxonomy, ecology and biotechnological potential. Front. Microbiol..

[9] Fusco V., Chieffi D., Fanelli F., Montemurro M. (2023). The *Weissella* and *Periweissella* genera: Up to date taxonomy, ecology, safety, biotechnological and probiotic potential. Front. Microbiol..

[10] Lakra A.K., Domdi L., Hanjon G., Tilwani Y.M., Arul V. (2020). Some probiotic potential of *Weissella confusa* MD1 and *Weissella cibaria* MD2 isolated from fermented batter. LWT.

[11] Fanelli F., Montemurro M., Verni M., Garbetta A., Bavaro A.R., Chieffi D., Cho G.-S., Franz C.M.A.P., Rizzello C.G., Fusco V. (2023). Probiotic Potential and Safety Assessment of Type Strains of *Weissella* and *Periweissella* Species. Microbiol. Spectr..

[12] Qiao N., Bechtner J., Cnockaert M., Depoorter E., Díaz-Muñoz C., Vandamme P., De Vuyst L., Gänzle M.G. (2023). Comparative genomic analysis of *Periweissella* and the characterization of novel motile species. Appl. Environ. Microbiol..

[13] Haiko J., Westerlund-Wikström B. (2013). The role of the bacterial flagellum in adhesion and virulence. Biology.

[14] Liu Q., Yu Z., Tian F., Zhao J., Zhang H., Zhai Q., Chen W. (2020). Surface components and metabolites of probiotics for regulation of intestinal epithelial barrier. Microb. Cell Fact..

[15] Shields P., Cathcart L. (2011). Motility Test Medium Protocol.

[16] Sørensen M.C., Gencay Y.E., Birk T., Baldvinsson S.B., Jäckel C., Hammerl J.A., Vegge C.S., Neve H., Brøndsted L. (2015). Primary isolation strain determines both phage type and receptors recognised by *Campylobacter jejuni* bacteriophages. PLoS ONE.

[17] Fanelli F., Montemurro M., Chieffi D., Cho G.S., Franz C.M.A.P., Dell’Aquila A., Rizzello C.G., Fusco V. (2022). Novel Insights into the Phylogeny and Biotechnological Potential of *Weissella* Species. Front. Microbiol..

[18] Tatusova T., DiCuccio M., Badretdin A., Chetvernin V., Nawrocki E.P., Zaslavsky L., Lomsadze A., Pruitt K.D., Borodovsky M., Ostell J. (2016). NCBI prokaryotic genome annotation pipeline. Nucleic Acids Res..

[19] Seemann T. (2014). Prokka: Rapid prokaryotic genome annotation. Bioinformatics.

[20] Afgan E., Baker D., Batut B., van den Beek M., Bouvier D., Čech M., Chilton J., Clements D., Coraor N., Grüning B.A. (2018). The Galaxy platform for accessible, reproducible and collaborative biomedical analyses: 2018 update. Nucleic Acids Res..

[21] Aziz R.K., Bartels D., Best A.A., DeJongh M., Disz T., Edwards R.A., Formsma K., Gerdes S., Glass E.M., Kubal M. (2008). The RAST Server: Rapid annotations using subsystems technology. BMC Genom..

[22] Cousin F.J., Lynch S.M., Harris H.M., McCann A., Lynch D.B., Neville B.A., Irisawa T., Okada S., Endo A., O’Toole P.W. (2015). Detection and genomic characterization of motility in *Lactobacillus curvatus*: Confirmation of motility in a species outside the *Lactobacillus salivarius* clade. Appl. Environ. Microbiol..

[23] Madeira F., Pearce M., Tivey A.R.N., Basutkar P., Lee J., Edbali O., Madhusoodanan N., Kolesnikov A., Lopez R. (2022). Search and sequence analysis tools services from EMBL-EBI in 2022. Nucleic Acids Res..

[24] Lemoine F., Correia D., Lefort V., Doppelt-Azeroual O., Mareuil F., Cohen-Boulakia S., Gascuel O. (2019). NGPhylogeny.fr: New generation phylogenetic services for non-specialists. Nucleic Acids Res..

[25] Letunic I., Bork P. (2021). Interactive Tree Of Life (iTOL) v5: An online tool for phylogenetic tree display and annotation. Nucleic Acids Res..

[26] Aizawa S.-I. (2014). The Flagellar World: Electron Microscopic Images of Bacterial Flagella and Related Surface Structures from More than 30 Species.

[27] Chen Y.F., Helmann J.D. (1995). The Bacillus subtilis flagellar regulatory protein sigma D: Overproduction, domain analysis and DNA-binding properties. J. Mol. Biol..

[28] Yokoseki T., Kutsukake K., Ohnishi K., Iino T. (1995). Functional analysis of the flagellar genes in the fliD operon of *Salmonella typhimurium*. Microbiology.

[29] Minamino T., Kinoshita M., Inoue Y., Morimoto Y.V., Ihara K., Koya S., Hara N., Nishioka N., Kojima S., Homma M. (2016). FliH and FliI ensure efficient energy coupling of flagellar type III protein export in *Salmonella*. Microbiologyopen.

[30] Neville B.A., Forde B.M., Claesson M.J., Darby T., Coghlan A., Nally K., Ross R.P., O’toole P.W. (2012). Characterization of Pro-Inflammatory Flagellin Proteins Produced by *Lactobacillus ruminis* and Related Motile Lactobacilli. PLoS ONE.

[31] Scharf B., Schuster-Wolff-Bühring H., Rachel R., Schmitt R. (2001). Mutational analysis of the *Rhizobium lupini* H13-3 and *Sinorhizobium meliloti* flagellin genes: Importance of flagellin A for flagellar filament structure and transcriptional regulation. J. Bacteriol..

[32] Tambalo D.D., Bustard D.E., Del Bel K.L., Koval S.F., Khan M.F., Hynes M.F. (2010). Characterization and functional analysis of seven flagellin genes in *Rhizobium leguminosarum* bv. viciae. Characterization of *R. leguminosarum* flagellins. BMC Microbiol..

[33] Mohari B., Thompson M.A., Trinidad J.C., Setayeshgar S., Fuqua C. (2018). Multiple Flagellin Proteins Have Distinct and Synergistic Roles in *Agrobacterium tumefaciens* Motility. J. Bacteriol..

[34] Faulds-Pain A., Birchall C., Aldridge C., Smith W.D., Grimaldi G., Nakamura S., Miyata T., Gray J., Li G., Tang J. (2011). Flagellin redundancy in *Caulobacter crescentus* and its implications for flagellar filament assembly. J. Bacteriol..

[35] Postel S., Deredge D., Bonsor D.A., Yu X., Diederichs K., Helmsing S., Vromen A., Friedler A., Hust M., Egelman E.H. (2016). Bacterial flagellar capping proteins adopt diverse oligomeric states. Elife.

[36] Wadhwa N., Sassi A., Berg H.C., Tu Y. (2022). A multi-state dynamic process confers mechano-adaptation to a biological nanomachine. Nat. Commun..

[37] Mondino S., San Martin F., Buschiazzo A. (2022). 3D cryo-EM imaging of bacterial flagella: Novel structural mechanistic insights into cell motility. J. Biol. Chem..

[38] Matsunami H., Barker C.S., Yoon Y.-H., Wolf M., Samatey F.A. (2016). Complete structure of the bacterial flagellar hook reveals extensive set of stabilizing interactions. Nat. Commun..

[39] Minamino T., Kinoshita M., Namba K. (2022). Insight into Distinct Functional Roles of the Flagellar ATPase Complex for Flagellar Assembly in *Salmonella*. Front. Microbiol..

[40] Cairns L.S., Marlow V.L., Kiley T.B., Birchall C., Ostrowski A., Aldridge P.D., Stanley-Wall N.R. (2014). FlgN is required for flagellum-based motility by *Bacillus subtilis*. J. Bacteriol..

[41] Kinoshita M., Hara N., Imada K., Namba K., Minamino T. (2013). Interactions of bacterial flagellar chaperone-substrate complexes with FlhA contribute to co-ordinating assembly of the flagellar filament. Mol. Microbiol..

[42] Minamino T., Kinoshita M., Morimoto Y.V., Namba K. (2021). The FlgN chaperone activates the Na^+^-driven engine of the *Salmonella* flagellar protein export apparatus. Commun. Biol..

[43] Minamino T., Kinoshita M., Hara N., Takeuchi S., Hida A., Koya S., Glenwright H., Imada K., Aldridge P.D., Namba K. (2012). Interaction of a bacterial flagellar chaperone FlgN with FlhA is required for efficient export of its cognate substrates. Mol. Microbiol..

[44] Kutsukake K., Okada T., Yokoseki T., Iino T. (1994). Sequence analysis of the *flg*A gene and its adjacent region in *Salmonella typhimurium*, and identification of another flagellar gene, flgN. Gene.

[45] Minamino T., Imada K. (2015). The bacterial flagellar motor and its structural diversity. Trends Microbiol..

[46] Mukherjee S., Kearns D.B. (2014). The structure and regulation of flagella in *Bacillus subtilis*. Annu. Rev. Genet..

[47] Capdevila S., Martínez-Granero F.M., Sánchez-Contreras M., Rivilla R., Martín M. (2004). Analysis of Pseudomonas fluorescens F113 genes implicated in flagellar filament synthesis and their role in competitive root colonization. Microbiology.

[48] Kalmokoff M., Lanthier P., Tremblay T.L., Foss M., Lau P.C., Sanders G., Austin J., Kelly J., Szymanski C.M. (2006). Proteomic analysis of *Campylobacter jejuni* 11168 biofilms reveals a role for the motility complex in biofilm formation. J. Bacteriol..

[49] Rabaan A.A., Gryllos I., Tomás J.M., Shaw J.G. (2001). Motility and the polar flagellum are required for *Aeromonas caviae* adherence to HEp-2 cells. Infect. Immun..

[50] Tsai J.-Y., Yeh Y.-H., Lin L.-D., Sun Y.-J., Hsiao C.-D. (2019). Crystal structure of the flagellin protein FlaG from *Helicobacter pylori*. J. Chin. Chem. Soc..

[51] Kurniyati K., Liu J., Zhang J.-R., Min Y., Li C. (2019). A pleiotropic role of FlaG in regulating the cell morphogenesis and flagellar homeostasis at the cell poles of *Treponema denticola*. Cell. Microbiol..

[52] Inoue T., Barker C.S., Matsunami H., Aizawa S.I., Samatey F.A. (2018). The FlaG regulator is involved in length control of the polar flagella of *Campylobacter jejuni*. Microbiology.

[53] Kajikawa A., Suzuki S., Igimi S. (2018). The impact of motility on the localization of *Lactobacillus agilis* in the murine gastrointestinal tract. BMC Microbiol..

[54] Lebeer S., Bron P.A., Marco M.L., Van Pijkeren J.P., O’Connell Motherway M., Hill C., Pot B., Roos S., Klaenhammer T. (2018). Identification of probiotic effector molecules: Present state and future perspectives. Curr. Opin. Biotechnol..

[55] Siciliano R.A., Mazzeo M.F. (2012). Molecular mechanisms of probiotic action: A proteomic perspective. Curr. Opin. Microbiol..

[56] Sharma R., Young C., Neu J. (2010). Molecular modulation of intestinal epithelial barrier: Contribution of microbiota. J. Biomed. Biotechnol..

[57] Troge A., Scheppach W., Schroeder B.O., Rund S.A., Heuner K., Wehkamp J., Stange E.F., Oelschlaeger T.A. (2012). More than a marine propeller—The flagellum of the probiotic Escherichia coli strain Nissle 1917 is the major adhesin mediating binding to human mucus. Int. J. Med. Microbiol..

