# Supplementary material for: Motility in Periweissella Species: Genomic and Phenotypic Characterization and Update on Motility in Lactobacillaceae
Source: Microorganisms. 2023 Dec 5;11(12):2923. doi: 10.3390/microorganisms11122923 (PMC10745875; doi:10.3390/microorganisms11122923)
Supplement: Supplementary file 1 [file microorganisms-11-02923-s001.zip › Supplementary Table S1_Strain list.pdf]

Supplementary Table S1. List of *Lactobacillaceae* type strains and WGS reference accession used in this study

genus	***species***	**type strain**	**Reference Accession**
Periweissella	*Periweissella beninensis*	LMG 25373^T	JAGMVS010000000
	Periweissella cryptocerci	26KH-42^T	NZ_CP037940.1
	Periweissella fabalis	LMG 26217^T	JAGMVU010000000
	Periweissella fabaria	LMG 24286^T	JAGMVV010000000
	Periweissella ghanensis	DSM 19935^T	JAGMVW010000000
Weissella	*Weissella bombi*	DSM 28794^T	NZ_FMAO00000000.1
	Weissella ceti	CCUG 59653^T	NZ_ANCA01000000.1
	Weissella cibaria	CCUG 41967^T	NZ_VNGZ01000000.1
	Weissella coleopterum	HDW19^T	NZ_CP049888.1
	Weissella confusa	ATCC 10881^T	NZ_CP027563.1
	Weissella diestrammenae	DSM 27940^T	JAGMVT010000000
	Weissella fangxianensis	HBUAS51963^T	JAMXDB000000000.1
	Weissella halotolerans	ATCC 35410^T	NZ_ATUU01000000.1
	Weissella hellenica	ATCC 51523^T	NZ_JAAXPM010000000.1
	Weissella jogaejeotgali	FOL01^T	NZ_CP014332.1
	Weissella kandleri	ATCC 51149^T	NZ_JQBP01000000.1
	Weissella koreensis	CCUG 47134^T	NZ_AKGG01000000.1
	Weissella minor	ATCC 35412^T	NZ_JQCD01000000.1
	Weissella muntiaci	8 H-2^T	NZ_SDGZ01000000.1
	Weissella oryzae	DSM 25784^T	NZ_DF820484.1
	Weissella paramesenteroides	ATCC 33313^T	ACKU01000000.1
	Weissella sagaensis	CCM 8924^T	BLKA01000001.1
	Weissella soli	CCUG 46608^T	NZ_CP017326.1
	Weissella thailandensis	CCUG 46557^T	NZ_BJEC01000000.1
	Weissella uvarum	B18NM42^T	JAGMVX010000000
	Weissella viridescens	ATCC 12706^T	NZ_CP061835.1
Lactobacillus	*Lactobacillus rogosae*	ATCC 27753^T	FONU01000000

Latilactobacillus	*Latilactobacillus curvatus*	DSM 20089[T]	AZDL00000000
Ligilactobacillus	*Ligilactobacillus acidipiscis*	DSM 15836[T]	AZFI00000000
	Ligilactobacillus agilis	DSM 20509[T]	AYYP00000000
	Ligilactobacillus ruminis	DSM 20403[T]	AYYL00000000
	Ligilactobacillus salitolerans	DSM 103433[T]	BFFP01000000
Liquorilactobacillus	*Liquorilactobacillus aquaticus*	DSM 21051[T]	AYZD00000000
	Liquorilactobacillus cacaonum	DSM 21116[T]	AYZE00000000
	Liquorilactobacillus capillatus	DSM 19910[T]	AZEF00000000
	Liquorilactobacillus ghanensis	DSM 18630[T]	AZGB00000000
	Liquorilactobacillus hordei	DSM 19519[T]	AZDX00000000
	Liquorilactobacillus mali	DSM 20444[T]	AYYH00000000
	Liquorilactobacillus nagelii	DSM 13675[T]	AZEV00000000
	Liquorilactobacillus oeni	DSM 19972[T]	AZEH00000000
	Liquorilactobacillus satsumensis	DSM 16230[T]	AZFQ00000000
	Liquorilactobacillus sucicola	DSM 21376[T]	AYZF00000000
	Liquorilactobacillus uvarum	DSM 19971[T]	AZEG00000000
	Liquorilactobacillus vini	DSM 20605[T]	AYYX00000000
