# Supplementary figures and images for: Motility in Periweissella Species: Genomic and Phenotypic Characterization and Update on Motility in Lactobacillaceae
Source: Microorganisms. 2023 Dec 5;11(12):2923. doi: 10.3390/microorganisms11122923 (PMC10745875; doi:10.3390/microorganisms11122923)

Tree scale: 0.1

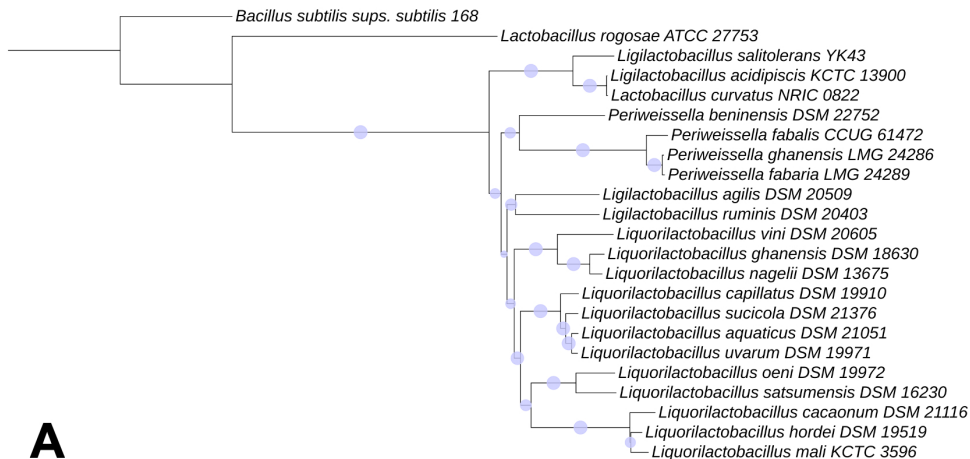

A

Tree scale: 1

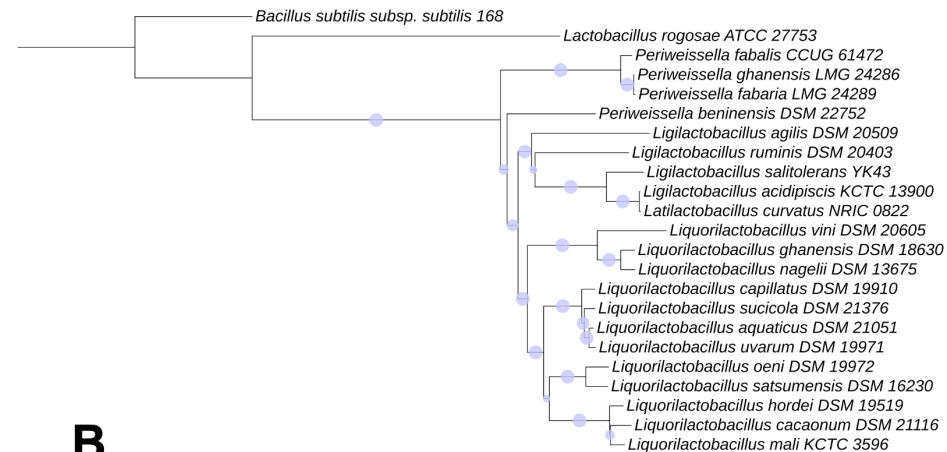

B

Tree scale: 1

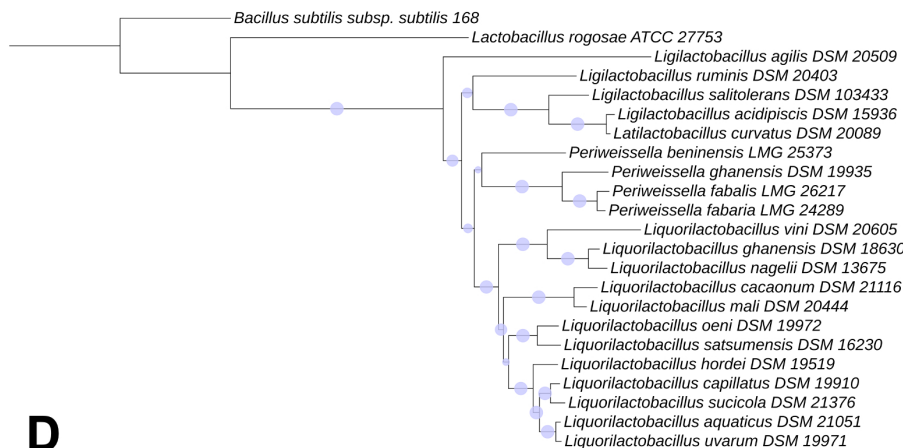

C

Tree scale: 1

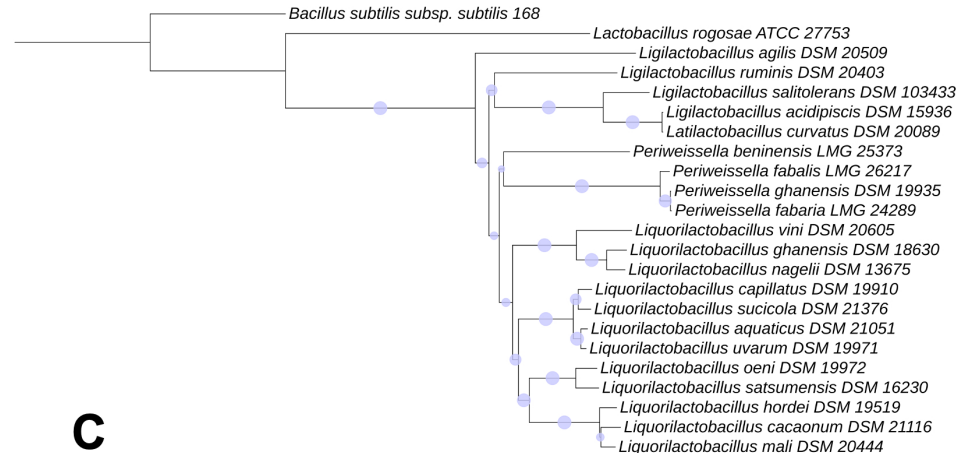

D

Supplement: Supplementary file 1 [file microorganisms-11-02923-s001.zip › Supplementary Figure S2_Phylogenetic tree motility proteins.pdf]
