# Supplementary material for: Motility in Periweissella Species: Genomic and Phenotypic Characterization and Update on Motility in Lactobacillaceae
Source: Microorganisms. 2023 Dec 5;11(12):2923. doi: 10.3390/microorganisms11122923 (PMC10745875; doi:10.3390/microorganisms11122923)
Supplement: Supplementary file 1 [file Supplementary Figure S1_FliI alignment.pdf › Supplementary Figure S1_FliI alignment.pdf]

```
FliI [P. beninensis]    1 MKFEISTDKYLTLAQQRKFFFTYGKVSEVVGLIIKVEGLDVFVGEICEVYIKGTQRKALT  60
FliI [P. fabalis]       1 MTFALTTAKYLNVVKQQPFTQTFGKVSEVVGLVIKVVGLASFVGEICEVHLTSNNKIALT  60
FliI [P. fabaria]       1 MTFALATDKYLTVVKQQPFTQTFGKVSEVVGLVIKVVGLASFVGEICEVHLASSHKVALT  60
FliI [P. ghanensis]     1 MTFALATDKYLTVVKQQPFTQTFGKVSEVVGLVIKVVGLASFVGEICEVHLASSHKVALT  60
Conservation
                          *7*4+7*5***9987*84*77*9*********9***4**56********8+588595***

                                     Walker B motif
FliI [P. beninensis]   61 EVVGFVKQTVLLMPLDELTRIGPGCLVKPTGHTLKVRVADALLGHTLDGLGRPLDDVTIT 120
FliI [P. fabalis]      61 EVVGFVKNTVLLMPLDDLEGISPGCLVVPTGRALTIKVNPALLGHTLDGLGRPMDDLEIT 120
FliI [P. fabaria]      61 EVVGFVKNTVLLMPLDDLDGISPGCLVVPTGRALTVKVNPALLGHTLDGLGRPMDDLQIT 120
FliI [P. ghanensis]    61 EVVGFVKNTVLLMPLDDLDGISPGCLVVPTGRALTVKVNPALLGHTLDGLGRPMDDLQIT 120
Conservation
                          *******9********9*64*8*****5***88*799*76*************9**96**

                                                                 Walker A motif
FliI [P. beninensis]  121 PDFKNYPVTQEAPDPFKRQKIKAIMPTGVRVIDGLMTVGEGQRMGIFAGSGVGKSTLLGM 180
FliI [P. fabalis]     121 DDFTEYSVMRDAPDPFKRPPIRETLPTGVRAIDGVLTVGNGQRMGIFAGSGVGKSTLLGM 180
FliI [P. fabaria]     121 EEFTEFPVMRDAPDPFKRPPIRETLPTGVRAIDGLLTVGNGQRIGIFAGSGVGKSTLLGM 180
FliI [P. ghanensis]   121 EEFTEFPVMRDAPDPFKRPPIRETLPTGVRAIDGLLTVGNGQRIGIFAGSGVGKSTLLGM 180
Conservation
                          59*7797*889*******74*9479*****8***99***7***9****************

FliI [P. beninensis]  181 IARYCAADVIVIGLIGERGREVKEFIEKDLGPEGYKKAVVVCATSDMPPLVRLKGAHVAT 240
FliI [P. fabalis]     181 MAKYAEADVIVIGLIGERGREVREFIEKDLGPEGYKKAVVVCATSDMPPLVRLKGAYVAT 240
FliI [P. fabaria]     181 MAKYADADVIVIGLIGERGREVREFIEKDLGPDGYKKAVVVCATSDMPPLVRLKGAYVAT 240
FliI [P. ghanensis]   181 MAKYADADVIVIGLIGERGREVREFIEKDLGPDGYKKAVVVCATSDMPPLVRLKGAYVAT 240
Conservation
                          9*9*94*****************9*********9**********************8***

FliI [P. beninensis]  241 AIAEYYRDAGKKVVLMMDSVTRFAMAQREIGLSTGEPPTTKGYTPSVFAALPKLLERSGM 300
FliI [P. fabalis]     241 AIAEYFRDQGKKVVLMMDSVTRFAMAQREIGLSTGEPPTTRGYTPSVFAMLPKLLERSGP 300
FliI [P. fabaria]     241 AIAEYFRDQGKKVVLMMDSVTRFAMAQREIGLSTGEPPTTRGYTPSVFAMLPKLLERSGP 300
FliI [P. ghanensis]   241 AIAEYFRDQGKKVVLMMDSVTRFAMAQREIGLSTGEPPTTRGYTPSVFAMLPKLLERSGP 300
Conservation
                          *****9**6*******************************9********8*********7

FliI [P. beninensis]  301 SDKGSITAFYTVLVEGDDMNEPIADAVRGILDGHIVLSRKIAGQNHYPAVDVQNSLSRLM 360
FliI [P. fabalis]     301 AEHGSITAFYTVLVEGDDMNEPIADAVRGILDGHIILSRKIAGQNHYPAIDVQSSLSRLM 360
FliI [P. fabaria]     301 AEHGSITAFYTVLVEGDDMNEPIADAVRGILDGHIILSRKIAGQNHYPAIDVQSSLSRLM 360
FliI [P. ghanensis]   301 AEHGSITAFYTVLVEGDDMNEPIADAVRGILDGHIVLSRKIAGQNHYPAIDVQSSLSRLM 360
Conservation
                          899********************************9*************9***9******

FliI [P. beninensis]  361 KSLVSKTQIQDASELRENLAVYADAKDLIDIGAYKAGSNAVIDYAISLNHPIDDFLQQQV 420
FliI [P. fabalis]     361 KSLVSPEHATAANKLRQSMATYNETKDLIEIGAYQIGTNQNVDQAINLHQPINQILTQSV 420
FliI [P. fabaria]     361 KSLVSPTHANAANKLRQSMATYNETKDLIEIGAYQVGTNQAVDHAINLHEPIKQILTQSV 420
FliI [P. ghanensis]   361 KSLVSPTHANAANKLRQSMATYNETKDLIEIGAYQVGTNQAVDHAINLHEPIKQILTQSV 420
Conservation
                          *****466785*97**899*8*798****9****77*8*669*6**9*55**578*8*8*

FliI [P. beninensis]  421 DEFANFEQTTDQLHQLFNGLSNL                                      443
FliI [P. fabalis]     421 DEHALFDNTVNQLKQLFQQPTMM                                      443
FliI [P. fabaria]     421 DEHAPFNNTVHQLEQLFQQPTMM                                      443
FliI [P. ghanensis]   421 DEHAPFNNTVHQLEQLFQQPTMM                                      443
Conservation
```
